# The Two-Spin Enigma: From the Helium Atom to Quantum Ontology

**DOI:** 10.3390/e26121004

**Published:** 2024-11-22

**Authors:** Philippe Grangier, Alexia Auffèves, Nayla Farouki, Mathias Van Den Bossche, Olivier Ezratty

**Affiliations:** 1 Laboratoire Charles Fabry, Institut d’Optique Graduate School, Centre National de la Recherche Scientifique, Université Paris Saclay, 91127 Palaiseau, France; 2 MajuLab International Joint Research Laboratory, Centre for Quantum Technologies, National University of Singapore, Singapore 117543, Singapore; alexia.auffeves@cnrs.fr; 3 Independent Researcher, 38054 Grenoble Cedex, France; naylafarouki@yahoo.fr; 4 Thales Alenia Space, 26, Avenue J.-F. Champollion, 31037 Toulouse, France; mathias.van-den-bossche@thalesaleniaspace.com; 5 EPITA Research Laboratory, 14-16 Rue Voltaire, 94270 Le Kremlin-Bicêtre, France; olivier@oezratty.net

**Keywords:** quantum physics, helium, electron spin, contextuality, operator algebra

## Abstract

The purpose of this article is to provide a novel approach and justification of the idea that classical physics and quantum physics can neither function nor even be conceived without the other—in line with ideas attributed to, e.g., Niels Bohr or Lev Landau. Though this point of view may contradict current common wisdom, we will show that it perfectly fits with empirical evidence, and can be maintained without giving up physical realism. In order to place our arguments in a convenient historical perspective, we will proceed as if we were following the path of a scientific investigation about the demise, or vanishing, of some valuable properties of the two electrons in the helium atom. We will start from experimentally based evidence in order to analyze and explain the physical facts, moving cautiously from a classical to a quantum description, without mixing them up. The overall picture will be that the physical properties of microscopic systems are quantized, as initially shown by Planck and Einstein, and that they are also contextual, i.e., they can be given a physical sense only by embedding a microscopic system within a macroscopic measurement context.

## 1. Prologue

*“Quantum mechanics occupies a very unusual place among physical theories: it contains classical mechanics as a limiting case, yet at the same time it requires this limiting case for its own formulation."* L.D. Landau and E.M. Lifshitz, *Quantum Mechanics* [1].

*“When you have eliminated all the impossible, then whatever remains, however improbable, must be the truth."* A. Conan Doyle, *The Case-Book of Sherlock Holmes* [2].

In 1923, Max Born gave a series of lectures at the University of Göttingen, from which he drew a monograph entitled *The Mechanics of the Atom* [3]. The second edition of this book was published in 1927 in English, just after the founding articles on quantum mechanics, and he wrote the following in the Preface:

*“Since the original appearance of this book in German, the mechanics of the atom has developed with a vehemence that could scarcely be foreseen. (⋯ However,) it seems to me that the time is not yet arrived when the new mechanics can be built up on its own foundations, without any connection with classical theory. It would be giving a wrong view of the historical development, and doing injustice to the genius of Niels Bohr, to represent matters as if the latest ideas were inherent in the nature of the problem, and to ignore the struggle for clear conceptions which has been going on for twenty-five years."*

As we now approach the hundredth anniversary of these historic discoveries, can we say, as Max Born intended, that *“the time is arrived when the new mechanics can be built up on its own foundations, without any connection with classical theory"*? The argument we want to develop here, and which—as Max Born also wanted—somehow does justice to the genius of Niels Bohr, is that this goal is in fact impossible to achieve. Indeed, based on a century of theoretical and experimental progress, our conclusion is that classical physics and quantum physics can neither function nor even be conceived without the other. Following the quotes above by Landau, Lifshitz, and Conan Doyle, this is a quite improbable situation indeed; nevertheless, it might well be the truth—we will see below why.

The general level of this article corresponds to that of an undergraduate physics course. First-year quantum mechanics students should easily follow the presentation, which deviates from textbooks more in the physical argumentation than in the technical content. Specific non-textbook terminology is introduced with bold font.

## 2. The Mysteries of Helium

The inability of classical physics to explain the structure of atoms, as well as the radiation they emit, is vividly illustrated in Max Born’s book, cited above. By adding to the classical equations the heuristic recipe of angular momentum quantification proposed by Niels Bohr, it is possible to compute the radiation—the spectrum—emitted by hydrogen and some other (so-called hydrogenoid) atoms. But these calculations spectacularly fail for helium, the simplest atom after hydrogen, with only two electrons—and Max Born’s book demonstrates that this is not due to a miscalculation by early-twentieth-century theorists. Mathematics is not magic, and correct calculations based on a physically false theory cannot provide results in accordance with experiments.

But other experiments and ideas appeared during the 1920s: the Stern–Gerlach experiment proposed by Otto Stern and carried out with Walther Gerlach in 1922; the hypothesis by George Uhlenbeck and Samuel Goudsmit in 1925 of an intrinsic angular momentum of the electron, called spin a little later by Wolfgang Pauli; and finally the exclusion principle, by Pauli again, applied first to electrons, which are particles with half-integer spin, as detailed in standard textbooks [1,4,5]. Finally, in 1925–26, the situation changed, as Max Born recounts in the previous section, and the solution appeared, *“with a vehemence that could scarcely be foreseen"*. The two electrons in the helium atom are actually two-spin 1/2 particles, which are strongly coupled by their electrostatic interaction and subject to the Pauli exclusion principle. It was understood that their state must be described mathematically by a so-called wave function, including their positions and spins, which must change sign (we say: be antisymmetric) by permutation of the two electrons. There are thus two families of states: those that are symmetric for the spatial part of the wave function, depending on the electrons’ positions, and antisymmetric for the spin; and those that are antisymmetric for the spatial part and symmetric for the spin.

Let us then focus on the lowest energy state of helium, called the ground state, which is symmetric for the spatial part and antisymmetric for the spin. The states of a spin 1/2 particle are traditionally denoted |+〉 and |−〉, corresponding to the measurement results +ℏ/2 and −ℏ/2 for the spin component along an arbitrary direction, often chosen along an Oz axis. The symbol ℏ=h/2π corresponds to a quantum of angular momentum, where $h\;≃\;6.63\;10^{-34}J.s$ is Planck’s constant. For two electrons we can thus define four states, |++〉, |+−〉, |−+〉, and |−−〉, where the first ± relates to one of the electrons and the second relates to the other. The corresponding values ±ℏ/2 could in principle be obtained from independent measurements on the two electrons. None of these four states changes sign by permutation of the two electrons, so none of them are suitable for the ground state of helium.

Then, in order to describe this state, we have to consider that in quantum formalism the symbol |ψ〉 for a quantum state, as introduced by Dirac [1,4,5], actually designates a vector in a mathematical sense: this means that linear combinations such as (a|+〉+b|−〉) for one spin or (c|++〉+d|+−〉+e|−+〉+f|−−〉) for two spins are other possible states, with the letters designating complex numbers. One can then see that the state $|s\rangle =(|+-\rangle -|+-\rangle )/\sqrt{2}$ provides a solution: by swapping the two electrons, we change |s〉 to −|s〉. This state |s〉 is called a ‘spin singlet’, we will see why later, and the calculation, taking |s〉 as the spin part of the electrons’ state, gives the correct values of the helium energy levels—a huge success for early quantum mechanics.

Now here is the enigma, which is the question that has painfully plagued physicists for 100 years: if the electron pair is in the state |s〉, what is the value of the projection component of the spin of the first electron along the Oz axis? And it unfortunately turns out that the formalism we have introduced above is completely incapable of answering this question. So let us be more concrete, and ask the following: in the |s〉 state, what value are we going to find by measuring the projection component of the spin of the first electron along the Oz axis? Then, there is an answer: we can randomly find either +ℏ/2 or −ℏ/2, with probabilities both equal to 1/2.

From here on, physicists tear themselves apart, as shown by many, often conflicting, interpretations on quantum mechanics which are documented in the Stanford Encyclopedia of Philosophy [6,7,8,9,10,11,12,13,14] or in [15]. Some physicists say that if we find +ℏ/2, it means that this projection was already worth +ℏ/2 before we measured it, because it cannot be otherwise, right? But some others answer that no, not at all, before the measurement the value of the projection was simply not defined—whatever it means. And still others say that, in fact, the measurement never happens; we have a branching of universes, one where the projection is +ℏ/2, and another where it is −ℏ/2. Still others claim that the result does not really exist, but that it is only a subjective bet made by the agent who made the measurement. And if we ask ‘but does the state |s〉 really exist’, some will say that this state may be ontic, epistemic, or epi-ontic⋯which does not really help.

It is thus clear that a terrible slippage has occurred along the way, and that we must take a few steps back and revisit the whole issue. In the line of our investigation, the enigma is then as follows: What caused the demise of the spin components of the individual electrons from our world? Did this really happen, and if yes, why and when? This question amounts to no less than clarifying what exists, what is real, and what is objective in a quantum phenomenon. And this is not mere philosophy: at the moment where quantum technologies bloom and concepts of quantum physics must be assimilated by engineers, these points have to be clarified—or more stuff that was thought to be real will keep on vanishing. As a matter of facts, engineers, like enquirers, do not work with metaphors that shed light on a deep but mysterious mathematical formalism. They need predictable and repeatable facts on which to build intuition and simple assessment rules because their action is at another complexity level, the one of building devices by having many elementary systems work together.

## 3. One Spin Is Almost Fine, Two Spins Are Really Weird

### 3.1. Further Investigations

**Figure 1:**
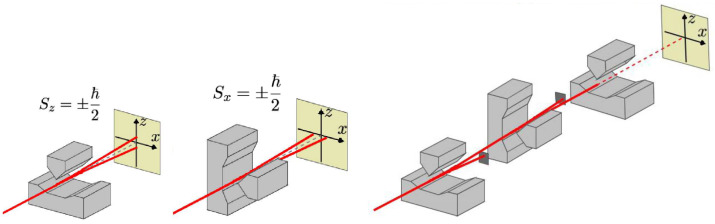
Stern–Gerlach magnets measuring the spin components along Oz (**left**), along Ox (**center**), and along Oz/Ox/Oz (**right**). In this last case, the results, −ℏ/2, are blocked by the dark square screens. © Manuel Joffre, Ecole Polytechnique.

Given the previous conundrum, it is appropriate to seek more expert advice, so let us refer to textbook quantum mechanics (TBQM) for more explanations [1,4,5]. In the previous part, we introduced the states |+〉 and |−〉, corresponding to the results +ℏ/2 and −ℏ/2 of a measurement of the projection of the electron’s spin in an Oz direction. Measuring the spin of an electron in an atom is not easy, but it can be conducted for atoms with a single ‘active’ electron. This is the principle of the Stern and Gerlach experiment we have already talked about, initially carried out with silver atoms. The Oz direction of the measurement corresponds to the direction of a magnetic field gradient that the atom experiences in the device, and must be chosen beforehand; a measurement along Oz does not tell what the result would be in another direction, Ox or Oy for example (see Figure 1). Nevertheless, repeating the same measurement along Oz will—not surprisingly—give the same result. So, from now on we will add a subscript indicating the measurement direction, such as $|\pm_{x}\rangle$ for Ox, and $|\pm_{z}\rangle$ for Oz.

We can then try to chain several measurements: first along Oz, which gives, let us say, +ℏ/2, then along Ox, then along Oz again. Theory and experiment say that the second measurement, along Ox, gives a random result ±ℏ/2, with probabilities of 1/2. Why not, so let us assume that we find +ℏ/2, and let us go back to the projection component along Oz that we knew before; but if we make the measurement again along Oz, we find a random result ±ℏ/2, with probabilities of 1/2: everything happens as if measuring along Ox had erased the previously known result along Oz ! So, already with a single-spin 1/2 particle, a strange vanishing of some properties can be observed—maybe a hint of what is going on with the more tricky two-spin situation.

Another very strange fact is this: suppose Alice randomly selects a particle in any one of the four states, $|\pm_{x}\rangle$ or $|\pm_{z}\rangle$, and sends it to Bob without any further indication. Bob is then unable to determine the state of the particle without error! Indeed, if he chooses (by chance) the same measurement axis as the one selected by Alice, he will find the right result, the one she has sent, but if he chooses another axis, he will find a random result, uncorrelated with what Alice sent. This impossibility, which also applies to any eavesdropper, is at the basis of the technique called quantum cryptography, or, more precisely, prepare-and-measure quantum key distribution. But then, how it is possible for Bob to obtain any useful information? This can be performed by Alice and Bob publicly exchanging their choice of axis after Bob has received the particle, and keeping only the results where they made the same choice; the revealed information is then useless for a possible eavesdropper, since the particle is no longer there [16].

The appearance of these random results raises several questions:

- Is it possible to find, in one way or another, situations where a measurement provides a deterministic result, i.e., one that can be predicted with certainty? This seems mandatory in order to attribute a physical reality to the objects we consider, and in the framework of our inquiry, we need actual and objective facts, not ghostly ones.
- If Bob cannot fully identify the state of the particle (the one known and sent by Alice) by making measurements of that particle, is it legitimate to say that |ψ〉 is the ‘complete state of the particle’?

Usual TBQM attributes a ‘complete quantum state’ to a particle, so its answer to the second question above is affirmative, but it also says that despite this completeness, Bob’s measurement result is generally not deterministic. Then, moving on to two particles, the contradictions accumulate, culminating in the strange situation mentioned at the end of the previous section. Therefore, unfortunately, one has to conclude that the expert advice from TBQM did not really help, but rather increased the confusion. So we will now look for different answers to these questions by saying that the quantum state may not be so complete after all—and show that we can thereby make one step towards elucidating this two-spin enigma, in helium and everywhere else.

### 3.2. Some Clues and First Answers to the Questions

In the rest of this article, we will reason about idealized experiments, in the sense that they are possible without contradicting anything we know about quantum mechanics (QM), but can be very difficult to perform in practice. Such reasoning has been used since the early days of QM, under the name of thought experiments, or Gedankenexperiments. In fact, it turns out that nowadays the progress in the mastery of quantum systems is such that a very large number of these thought experiments have been carried out, with such a good degree of approximation that they can be considered to experimentally validate the reasoning initially carried out in principle.

This is particularly the case for what are now called ‘Quantum Non-Demolition measurements’, or QND measurements, in which the quantum state of a particle is identified while leaving that particle in the observed state afterwards [17]. These measurements are sometimes referred to as von Neumann’s ideal measurements, because John von Neumann used them extensively from a theoretical point of view [18]; but it is now possible to consider them well-established experimental facts [19,20,21]. They have the considerable advantage of giving a clearly positive answer to our first question, i.e., whether it is possible to obtain, in one way or another, deterministic results. The answer is yes, because it is enough to repeat a QND measurement on the same particle or system to find the same result again with certainty. Let us insist on the essential point that it is not only a question of chaining together different measurements on the same system, which leads to random results as we have written before: it is a question of repeating the same measurement on the same system. This certainty may not be as general as we might have liked, because it is destroyed by making a different measurement, as said before, but it is consistent with what we learn in QM textbooks and observe in the lab, so it will allow us to move forward.

The answer to the second question, ‘if Bob cannot find the state of the particle by measuring that particle, is it legitimate to say that it is indeed the state of the particle?’ is going to take us further off the beaten track. We have already seen that if Alice gives Bob the particle without indicating its state, Bob cannot return to that state with certainty. On the other hand, if Alice gives Bob the particle without indicating its state, but also indicating the direction of measurement she used, Ox for example, then Bob can identify with certainty which state, $|{+}_{x}\rangle$ or $|{-}_{x}\rangle$, was prepared by Alice. We thus find the same certainty as before, provided again that the correct measurement is repeated, i.e., that Alice’s and Bob’s axes agree. Introducing a bit of terminology, we will call the set of classical parameters defining Bob’s action a ‘measurement context’, or, more briefly, a **context** denoted by C and materialized by a macroscopic classical device. We can thus say that the physical object that owns a certain and reproducible measurement result is not the system (the particle) alone, but the system within a context. In this framework, **the possible results of a specified measurement, attributed to a system within a context, are called modalities** [22].

If Bob has the quantum particle and knows the context used by Alice to prepare the particle, then he can identify an associated modality. For a single spin, the context is defined by the direction of the projection, Oz for example, and there are only two mutually exclusive results, ±ℏ/2. A possible modality is then +ℏ/2 along Oz, corresponding to the vector $|{+}_{z}\rangle$ introduced earlier. For a single spin, it seems that there is no difference between a state and a modality, but we will see later that the distinction between the usual (textbook) state vector and the modality becomes essential for larger systems, e.g., for two spins.

To summarize this section, we have found some determinism in the behavior of the physical objects used in QM, if we accept that they are in fact (quantum) systems within (classical) contexts. Then, the very existence of the victim of the demise—the spin component of the electron—may become uncertain indeed, because it is no longer a property belonging to the electron alone. Clearly, the modality may change if we change the context—and a new modality will show up in a new context, and will be then certain and repeatable. But before exploiting this major result in our inquiry, let us return once again to helium, and more precisely to the other family of helium states, those that are antisymmetric for the spatial part and symmetric for the spin—that is, their spin state does not change its sign by permutation of the two electrons.

### 3.3. The Solution to the Enigma—Or Not?

The four possible states, $|{+}_{z}{+}_{z}\rangle$, $|{+}_{z}{-}_{z}\rangle$, $|{-}_{z}{+}_{z}\rangle$, and $|{-}_{z}{-}_{z}\rangle$, for the two spins correspond to separate measurements made on the two electrons, and do not all fit with the symmetry or antisymmetry condition imposed by the Pauli exclusion principle. We have already seen that the vector $|s\rangle =(|{+}_{z}{-}_{z}\rangle -|{-}_{z}{+}_{z}\rangle )/\sqrt{2}$, the singlet state, is antisymmetric. But what are the symmetric states? One can see that $|{+}_{z}{+}_{z}\rangle$ and $|{-}_{z}{-}_{z}\rangle$ are suitable, as well as the state $|r\rangle =(|{+}_{z}{-}_{z}\rangle +|{-}_{z}{+}_{z}\rangle )/\sqrt{2}$: we therefore have three symmetric states, globally called triplet states.

**Figure 2:**
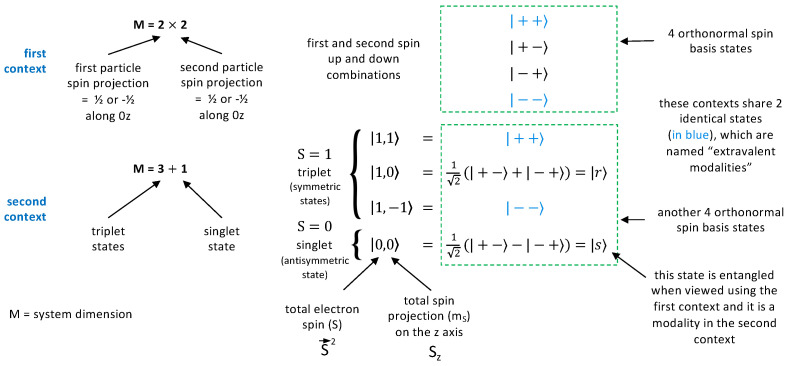
Various quantum states relevant for two electrons. All |±〉 mean $|\pm_{z}\rangle$. The total spin ${\vec{S}}^{2}$ is the sum of the squares of the three spin projections $\left(S_{x}^{2}+S_{y}^{2}+S_{z}^{2}\right)$ and it takes the values $S\left(S+1\right)\hbar^{2}$. In this figure, *M* is the total number of possible, mutually exclusive measurement outcomes—or modalities—in each context.

We have already said that the symbols |ψ〉 are vectors; now, it must be specified that the four vectors $|\pm_{z}\pm_{z}\rangle$ are in fact orthogonal, and correspond to measurement results, modalities as defined above, which are mutually exclusive: only one of these four results can be obtained when measuring the components along Oz of the spin of each electron. Let us then consider the four states, $|{+}_{z}{+}_{z}\rangle$ and |s〉, |r〉, $|{-}_{z}{-}_{z}\rangle$. An elementary calculation shows that they are also orthogonal, and thus also describe mutually exclusive measurement results, but what is the associated measurement? A standard algebraic calculation in QM shows that this is the measurement of the quantities ${\vec{S}}^{2}$ and $S_{z}$, where $\vec{S}$ is the total spin of the two electrons $\vec{S}={\vec{S}}_{1}+{\vec{S}}_{2}$; it is associated with the two quantum numbers S=0 and S=1 (see Figure 2). For S=0, a measurement of the total spin along Oz (or an arbitrary axis) gives the value 0: this is in fact the state |s〉. For S=1, a measurement of the total spin along Oz gives the values +ℏ for the state $|{+}_{z}{+}_{z}\rangle$, 0 for the state |r〉, and −ℏ for the state $|{-}_{z}{-}_{z}\rangle$. So, we again have four mutually exclusive results, which are noted |S=0,m=0〉, |S=1,m=1〉, |S=1,m=0〉, and |S=1,m=−1〉, where *m* is the result mℏ of the measurement. We note also that we can write $\left|S=1,m=1\rangle =\right|{+}_{z}{+}_{z}\rangle$ and $\left|S=1,m=-1\rangle =\right|{-}_{z}{-}_{z}\rangle$: these are therefore the same ‘state vectors’, but not the same modalities, since these results appear in completely different measurement contexts, corresponding either to measurements on the separate spins or to a global measurement of the total spin. This distinction is quite important [23], and we will return to it later; see Figure 2 for a summary.

Though the inquiry is clearly making progress, we will conclude this part with a new conundrum by again considering the states/modalities |s〉 and |r〉, perfectly defined for a measurement of the total spin, and associated with well-defined energy levels of the helium atom. As we have already seen, the question ‘what is the value of the component of the spin along Oz for only one of the two electrons’ has no answer within the framework of the formalism of usual QM we have introduced. We can reconcile ourselves with this statement by saying that of course there is no answer, but this is not really shocking since we are talking about two electrons within the same atom, very strongly coupled by electrostatic interaction, so it is not very surprising that they adopt a global behavior. In addition, there is no clear way to measure each electron’s spin within the helium atom.

Yet the ever-doubting physicist will ask the following question: ok, but can’t we have the two electrons, or any two spin 1/2 particles, very far apart, and still in the state |s〉 or in the state |r〉? This can be conducted in suitable experiments, as detailed in the Appendix A. What, then, is the meaning of the statement that the component of its spin along Oz has no value, or a completely random value? And what happens if we compare the results of separate measurements on the two spins? Then, we have to dive into an even deeper mystery, that of quantum entanglement, Verschränkung in German, with two famous papers published in 1935: ‘Can Quantum-Mechanical Description of Physical Reality Be Considered Complete?’, by Einstein, Podolsky, and Rosen (EPR) [24], and Schrödinger’s almost desperate article, though resigned to the quantum weirdness, ‘Die gegenwärtige Situation in der Quantenmechanik (The present situation in quantum mechanics)’ [25]. The cause of desperation of these great physicists was the following: the spin components of the two remote electrons, which were supposed to have “no value”, turn out to be strongly correlated! But how can it be, if their values are undetermined? Did QM fall from Charybdis to Scylla? It is this new extended enigma that will now have to be solved.

## 4. Predictive Incompleteness of |ψ〉 with No Context

### 4.1. The Bell Perspective

The problems raised by Einstein and his colleagues in 1935 addressed the almost philosophical foundations of QM but had no direct experimental implications. The situation changed in 1964 when John Bell proposed inequalities, deduced from hypotheses very close to those discussed by EPR, and which could be tested experimentally on entangled pairs of particles [26]. This stimulated a long series of experiments, starting in 1972, with highlights in 1982 and 2015 [27], that fortunately concluded with the Nobel Prize awarded in 2022 to Alain Aspect, John Clauser, and Anton Zeilinger ‘for experiments with entangled photons, establishing the violation of Bell’s inequalities and pioneering quantum information science’. We are not going to tell this story here, since it has been the subject of many articles and books, but rather consider the following question: what is ultimately the content of Bell’s hypotheses, leading to these inequalities, and now dismissed by observations? Many possible answers have been proposed, but we will now develop one that is in line with the content of the previous sections.

A possible understanding of Bell’s perspective says that regardless of the limitations of actual measurements, the underlying reality should have a definite configuration, described by some local hidden variables (LHVs), that fully specifies everything that can/will happen. The existence of a physical mechanism producing an effect from LHVs as a cause is very generally true in classical physics, and it is also built on the use of classical probability theory. It means that even if the values of the LHV are not known, they are supposed to exist as the ‘hidden cause’. Such an explanatory mechanism is generally called the completeness of inference, or **predictive completeness**, given the hidden parameters [28]. Let us emphasize that this predictive completeness does not mean fully knowable determinism but ontological determinism, i.e., the existence of a fundamental underlying predictable mechanism, as expressed by Laplace’s demon or by Einstein’s famous quote “God does not play dice”. What is at stake is thus the possibility of specifying everything that will happen, in a classically deterministic or at least in a probabilistic theory, which is also assumed by Bell to be local. Equivalently, one may say that randomness has an “ignorance interpretation”: like in tossing a coin, the result is random, but in principle knowing all the initial parameters and the laws of dynamics should allow one to predict the result with certainty.

Now, without further ado or mathematics, it is clear that the usual quantum state vector, as introduced in the previous section for describing the spin systems and generically denoted by |ψ〉, does not fit in this framework. Specifying everything that may happen can only be performed once the measurement context has been specified. More precisely, this measurement context is required, in addition to |ψ〉, in order to define a standard probability distribution over a set of mutually exclusive events. In this sense, we can say that for the spin systems the quantum state alone is **predictively incomplete**. Specifying all these possible events (i.e., the possible measurement results) is equivalent to giving the context, and |ψ〉 allows one to calculate their respective probabilities of occurring. Since this is obtained by specifying both |ψ〉 and the context, we have to acknowledge that what QM allows are **contextual inferences** [28].

Though this just tells how quantum mechanics works, it may appear shocking to both a classical physicist and a (naive) quantum physicist. A classical physicist has a hidden cause of randomness and correlations (Bell’s LHV) deeply rooted in their mind, so saying that there are no such variables in quantum physics is hard to accept. On the other hand, a quantum physicist has deeply rooted in their mind that |ψ〉 is complete, in the sense that there is no underlying hidden cause to be sought—as it was taught by Niels Bohr, and as it is still true. But saying that the quantum state vector |ψ〉 is incomplete as long as the context has not been specified, though it is operationally obvious in quantum mechanics, and does not contradict Bohr’s message, may be quite hard to accept. Why? Because we would have then to give up the idea that |ψ〉 fully specifies a physical object, which is the quantum system under study, and to admit that **the actual physical object has to be the quantum system within a classical context.** This appears so shocking that the quantum physicist may be tempted to consider instead ‘non-local influences’, or other weird explanations, in order to violate Bell’s inequalities; but this leads to the contradictory points of views presented before, and it is actually not needed if predictive incompleteness is acknowledged [28].

### 4.2. Mutual Consistency of the Classical and Quantum Descriptions of the Physical World

The idea that context is needed to obtain a complete description of what can be observed on a quantum system has often been confused with statements about the subjectivity of the quantum state, or about the role of the consciousness of the observer [15]. There is no need for that either, and a physical object remains perfectly objective, but it is still a system within a context. This requires one, however, to recognize that there is no such thing as absolute objectivity of predictions about the system alone; this departs from the usual, classical, apprehension of the universe, taken as a whole, with all its logical and ontological necessity as Newton and Einstein conceived it. The quantum requirement for a contextual description of physical objects is related to many issues in quantum physics, and it has been spelled out initially in the article ‘Contextual objectivity: a realistic interpretation of quantum mechanics’ (2001) [29], which may provide us with a more suitable, non-classical view about the world we live in [30].

There is clearly a price to pay for the idea that there are well-defined systems and contexts: if this is the case, there must be a physical separation between them, usually known as the “Heisenberg cut”, where the applicable laws change from quantum to classical. This creates a dualist view of the physical world that does not seem satisfactory, and there have been many attempts to find ways by which the classical world should ‘emerge’ from the quantum one—if any. This was also the content of Max Born’s initial request, to build up ‘the new mechanics on its own foundations, without any connection with classical theory’. But it can also be said, as Bohr and Landau did, that there is no way to formulate quantum mechanics without referring to classical concepts. For instance, Landau and Lifshitz [1] write *“It is clear that, for a system composed only of quantum objects, it would be entirely impossible to construct any logically independent mechanics (⋯) Thus quantum mechanics occupies a very unusual place among physical theories: it contains classical mechanics as a limiting case, yet at the same time it requires this limiting case for its own formulation”*.

This leads to the idea that **classical and quantum descriptions are both required from the beginning and are simultaneously needed for mutual consistency** [31]. Actually, classical descriptions without quantum descriptions are unable to explain the structure of atoms, as seen above, but quantum descriptions without classical descriptions are unable to say clearly what a quantum state describes, and when extrapolated to the macroscopic world they become lost in bizarre predictions that clash with empirical evidence.

An apparent difficulty for unifying classical and quantum physics is that their mathematical frameworks seem incompatible – but this is not correct, and a unified formalism, using operator algebras, is actually possible and was already suggested by John von Neumann in 1939. Though this extension of the standard quantum formalism is mathematically nontrivial, it definitively deserves a closer look by quantum physicists—after being placed into the appropriate ontological framework introduced above. This has been discussed in several recent articles [32].

### 4.3. Recovering Real Properties: Modalities vs. State Vectors

An important step in our discussion has been to introduce a specific word representing a physical property or set of physical properties, attributed to a system within a context—we call it a modality. Clearly, a modality is different from a usual quantum state vector |ψ〉, which does not specify the context. It is important to note that for a single-spin 1/2 particle, just giving a quantum state vector like $|{+}_{z}\rangle$ actually specifies the context Oz, and there is only one other mutually exclusive modality, that is, $|{-}_{z}\rangle$. But for larger values of the spin, or for more particles, there are more than two mutually exclusive modalities in a given context. In that case, a given quantum state vector may appear in infinitely many contexts, as was the case (for two different contexts) with $|{+}_{z}{+}_{z}\rangle =|S=1,m=1\rangle$ in the example given above.

One should also note that the quantum state vectors $|\pm_{z}\rangle$ and $|\pm_{x}\rangle$ are related by $|{+}_{x}\rangle =(|{+}_{z}\rangle +|{-}_{z}\rangle )/\sqrt{2}$ and $|{-}_{x}\rangle =(|{+}_{z}\rangle -|{-}_{z}\rangle )/\sqrt{2}$: the state vectors along Ox are linear superpositions of the state vectors along Oz. It is often said that ‘quantum superposition are like being in the two states $|{+}_{z}\rangle$ and $|{-}_{z}\rangle$ at the same time’, which clearly makes no sense: the modalities associated with $|\pm_{x}\rangle$ are certainties in the Ox context, mathematically expressed as linear combinations of state vectors $|\pm_{z}\rangle$ that are certainties in the Oz context. It is also useful to keep in mind that the state |s〉 appears entangled in the context where the two spins are described and measured separately, but not in the context based on the total spin with S=1 or S=0. Entanglement is thus related to the choice of a description and measurement context (technically, a tensor product structure) for the system of interest. Obviously, the description using |±,±〉 appears more ‘natural’ when the two spins are spatially separated.

Putting everything together, the theoretical tools used so far consist of associating orthogonal vectors with mutually exclusive modalities (in the same context), and the same vector with mutually certain modalities (in different contexts). Introducing some vocabulary, the non-obvious physical phenomena that certainty can be transferred between contexts is called **extracontextuality** [23]. Then, the property of ‘being mutually certain’ defines an equivalence relation between modalities, which is called **extravalence**—think again of the relation $|{+}_{z}{+}_{z}\rangle =|S=1,m=1\rangle$.

We come thus to the conclusion that **the quantum state vector |ψ〉 is a mathematical object associated with an extravalence class of modalities.** This establishes a clear separation between mathematical objects like |ψ〉 and physical objects that are systems within contexts and carry well-defined and measurable properties that correspond to modalities. These sentences require some thinking to be fully appreciated, but they do refer to the ontology of physical objects (systems and contexts) and their properties (modalities) within the non-classical but realistic framework provided by contextual objectivity.

To complete the picture, we need a fundamental postulate, called contextual quantization, which says that **the maximum number *M* of mutually exclusive modalities is a property of the quantum system, and it is the same in any relevant context.**

We have already seen an example of this behavior with the two electron spins, where *M* = 4 for several possible contexts—this is actually the case for any such context. Given this postulate, it can be shown that the relation between modalities in different contexts can only be probabilistic [33]. The fixed value of *M* also allows one to associate the system with an *M*-dimensional vector space—actually a Hilbert space [34]. In this picture, there is no trouble in using the standard projection postulate of textbook QM, which appears simply as an update of the probability distribution associated with |ψ〉, for a given context.

The crucial point is that this probability distribution is non-classical and contextual, and it becomes a computable physical meaning only once the actual context has been specified [33]. For the two-spin enigma, the violation of Bell’s inequalities is attributed to the predictive incompleteness of |ψ〉, and it does not require any non-local influence, but rather a contextual inference, as explained in detail in [28]. So, there are no ‘hidden variables’ to be looked for, and the context, completing |ψ〉, specifies the “very conditions which define the possible types of predictions regarding the future behavior of the system”, as stated by Bohr [35]. Mixing up probabilities calculated in different contexts is counterfactual and leads to contradictions. There is no doubt that these explanations are quite remote from classical physics, but again they make full sense in the framework of contextual objectivity.

## 5. Epilogue

Since the purpose of this paper is to illustrate and discuss quantum ideas using two-spin 1/2 particles, we did not reconstruct the full quantum formalism yet, and explanations of basic tools like unitary transforms and Born’s rule are still missing. But there is not much choice left, because the framework defined above fits with the hypotheses of powerful mathematical theorems, establishing the need for unitary transformations (Gerhard Uhlhorn’s theorem [36]) and for Born’s rule (Andrew Gleason’s theorem [37]). These arguments have been published elsewhere [34] and will not be reproduced here. Also, systems and contexts should be part of a unified mathematical formalism, going beyond standard textbook QM. Operator algebra, introduced by Murray and von Neumann [38,39] and considerably developed since then, can provide such a formalism and give a mathematical status to the Heisenberg cut, as has been shown in [32].

So finally, what should we say to the layman to bring our investigation to a successful conclusion and to replace misleading statements such as “a quantum superposition is like being in two states at the same time”, or “quantum entanglement is like an instantaneous action at a distance”? It could be the following:

*The physical properties of microscopic systems are quantized, as initially shown by Planck and Einstein, and they are also contextual; this means that they can be given a physical sense only by embedding a microscopic system within a macroscopic context that specifies how the system is observed, as proposed by Bohr. Such a behavior is quite remote from the non-quantized and non-contextual perspective of classical physics, and it requires developing a specific contextual probabilistic theory, which is basically what quantum mechanics has been doing.*

To conclude, let us emphasize that QM follows from the conjunction of quantization and contextuality; these two properties taken separately may have some classical analogue, and therefore they do not have the same constraining power when being combined.

## Figures and Tables

**Figure A1:**
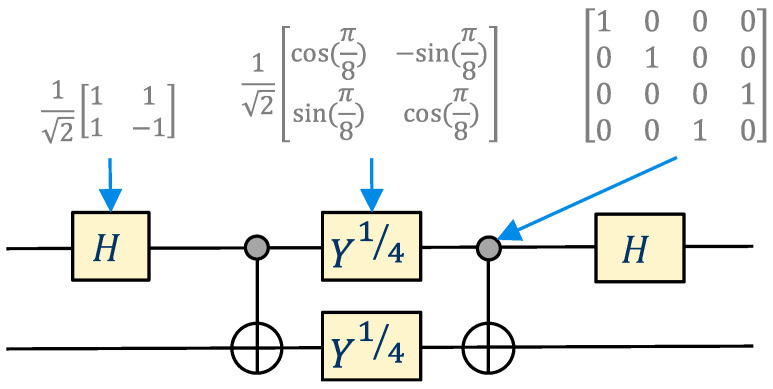
Sequence of one- and two-qubit quantum gates and their matrix unitaries acting on two qubits, to move between the coupled and uncoupled basis. The first and last gates are Hadamard gates, the second and forth Controlled-NOT gates, and the middle ones are specific non-Clifford one-qubit gates.

## Data Availability

This is theoretical research, so no new data were created.

## Appendix A. Another View on Two-Spin 1/2 Particles

In the above text, we use the example of the helium atom to introduce the four states $|{+}_{z}{+}_{z}\rangle$, |r〉, |s〉, and $|{-}_{z}{-}_{z}\rangle$, which can also be written as |S=1,m=1〉, |S=1,m=0〉, |S=0,m=0〉, and |S=1,m=−1〉. In the helium atom, these states are imposed by the Pauli exclusion principle and can be identified because they correspond to specific energy levels. On the other hand, the context corresponding to the separated states of the two electrons, $|{+}_{z}{+}_{z}\rangle$, $|{+}_{z}{-}_{z}\rangle$, $|{-}_{z}{+}_{z}\rangle$, and $|{-}_{z}{-}_{z}\rangle$, is not accessible within the helium atom, since this would require one to have individual access to the electrons by taking them far away from the atom and thus changing their energy state.

So, it would be nice to exhibit another system, where all these states can be manipulated and measured. This has been proposed by photo-dissociating a Hg_2_ molecule in a singlet state and carrying out spin measurements on the two remote fragments [40]. But this turned out to be a quite difficult experiment, and another more feasible approach is to consider that a spin 1/2 particle is actually a quantum bit, a qubit in a quantum computer, and identify $|{+}_{z}\rangle$ with the logical state |1〉 and $|{-}_{z}\rangle$ with the logical state |0〉.

The question is then to move from the four states (‘uncoupled basis’) |1,1〉, |1,0〉, |0,1〉, and |0,0〉, usually called the computational basis, to the four states (‘coupled basis’) |1,1〉, $(|1,0\rangle +\left|0,1\rangle \right)/\sqrt{2}$, $(-|1,0\rangle +\left|0,1\rangle \right)/\sqrt{2}$, and |0,0〉. This can be carried out by the following 4×4 unitary matrix:

$$G=\left(\begin{array}{cccc} 1 & 0 & 0 & 0\\ 0 & 1/\sqrt{2} & -1/\sqrt{2} & 0\\ 0 & 1/\sqrt{2} & 1/\sqrt{2} & 0\\ 0 & 0 & 0 & 1 \end{array}\right)$$

which can be implemented by a sequence of quantum gates, as shown in Figure A1. This shows explicitly that, as written in the text, two qubits can be set or measured in the state |s〉 or in the state |r〉 outside the helium atom.

An arbitrary quantum state can thus be measured in either the computational basis {|1,1〉, |1,0〉, |0,1〉, |0,0〉} or in the coupled basis {|1,1〉, $(|1,0\rangle +\left|0,1\rangle \right)/\sqrt{2}$, $(-|1,0\rangle +\left|0,1\rangle \right)/\sqrt{2}$, |0,0〉}. More precisely, the nontrivial measurement in the coupled basis can be realized by applying $G^{†}$ to the two qubits, carrying out a QND measurement in the uncoupled (computational) basis, which is in principle easy, and then applying *G* to recover the measured state in the coupled basis. Therefore, such a given sequence of gates, building up a unitary transformation, implements a controlled change of context, since not only one but a whole set of mutually orthogonal states are mapped onto another set of mutually orthogonal states. This has the advantage that modalities can be defined up to a unitary transform, in the case where the appropriate context cannot be implemented. Though not so easy to implement in practice, this two-way procedure ($G^{†}$/QND/*G*) is useful in quantum computing and shows that thinking about physical modalities, associated with a context, may be more enlightening than just thinking about mathematical state vectors, which represent extravalence classes of modalities occurring in different contexts.
